# Supplementary material for: Complexome profiling of the Chlamydomonas psb28 mutant reveals TEF5 as an early PSII assembly factor
Source: Plant Cell. 2025 Mar 17;37(6):koaf055. doi: 10.1093/plcell/koaf055 (PMC12164590; doi:10.1093/plcell/koaf055)
Supplement: koaf055_Supplementary_Data [file koaf055_supplementary_data.zip › psb28_interactive.zip!/plots/intensity/qualityPlots/peptides/_Cre01.g049350.t1.1.html]

### Cre01.g049350.t1.1

Trivial name:   
SeqCoverage [median]: 3.60  
Mass: 53.06 kDa  
  
 

| Sequence | MissedCleavage | Mass [Da] | wtIntensity | muIntensity | Score |
| --- | --- | --- | --- | --- | --- |
| GLDTQISR | 0 | 888.47 | 2442.76 | 9706.03 | 122.52 |
| GRDPAVSYQK | 1 | 1119.57 | 2525.69 | 8351.05 | 66.49 |
|  |  |  | 4968.4 | 18057.1 |  |
